# Supplementary material for: Unveiling the co-phylogeny signal between plunderfish Harpagifer spp. and their gut microbiomes across the Southern Ocean
Source: Microbiol Spectr. 2024 Mar 5;12(4):e03830-23. doi: 10.1128/spectrum.03830-23 (PMC10986581; doi:10.1128/spectrum.03830-23)
Supplement: Supplemental material — Figures S1 to S3 and Tables S1 to S5. [file spectrum.03830-23-s0001.docx]

**Unveiling the co-phylogeny signal between plunderfish *Harpagifer* spp. and their gut microbiomes across the Southern Ocean**

**Running title**

Phylosymbiosis and co-phylogeny in *Harpagifer*

**Authors**

Guillaume Schwob^1,2,3#^, Léa Cabrol^1,3,4^, Thomas Saucède^5^, Karin Gérard^1,6,7^, Elie Poulin^1,2,3^ and Julieta Orlando^1,2^

**Authors’ affiliations**

^1^Millennium Institute Biodiversity of Antarctic and Subantarctic Ecosystems (BASE), Santiago, Chile

^2^Department of Ecological Sciences, Faculty of Sciences, University of Chile, Chile

^3^Institute of Ecology and Biodiversity, Santiago, Chile.

^4^Aix-Marseille University, Mediterranean Institute of Oceanography (M.I.O, UMR 110), CNRS, IRD, Marseille, France

^5^UMR 6282 Biogeosciences, Univ. Bourgogne Franche-Comté, CNRS, EPHE, Dijon, France

^6^Laboratory of Antarctic and Subantarctic Marine Ecosystems, Institute of Patagonia, University of Magallanes, Punta Arenas, Chile.

^7^Cape Horn International Center, Puerto Williams, Chile

^#^corresponding author: gschwob@institutobase.cl

**Supplemental material**

**Supplementary Figure 1.** **Redundancy analysis (RDA) ordination of the sampling localities based on a set of seawater properties collected in silico from the Bio-ORACLE database (v2.1).**

Dots are colored according to the sampling locality. The colored vectors indicate the contribution of each seawater variable on the two PCA dimensions. All these properties are mean values estimated at mean depth. Temp_range: Range of the seawater temperature, Temp_max: Maximum temperature, Temp_min: Minimum temperature, Temp_mean: Mean temperature, Chlo_mean: Chlorophyll A concentration, Carbonphyto_mean: mole concentration of phytoplankton expressed as carbon, pp_mean: net primary productivity of carbon, Nitrate_mean: mole concentration of nitrate, Salinity_mean: Salinity, Dissox_mean: mole concentration of dissolved molecular oxygen, Phosphate_mean: mole concentration of phosphate, Silicate_mean: mole concentration of silicate, Iron_mean: Mean mole concentration of dissolved iron.

**Supplementary Figure 2.** **Divergence of *Harpagifer* populations across sampled biogeographic regions.**

Median-joining haplotypes network computed from COI sequences of *Harpagifer* sp.


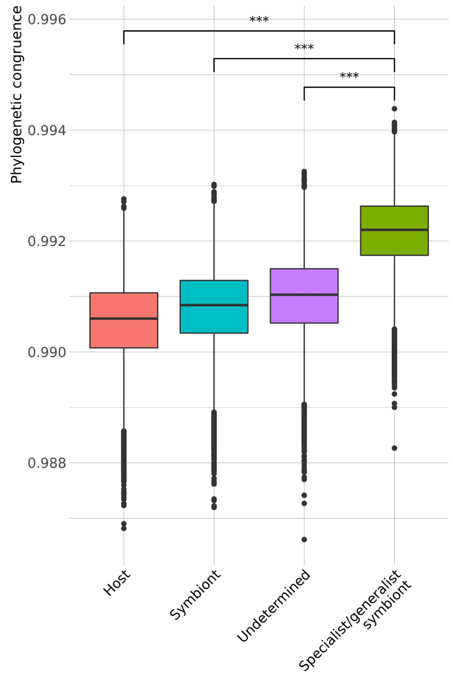
**Supplementary Figure 3. Phylogenetic congruence between *Harpagifer* hosts and *Aliivibrio* according to the randomization algorithms implemented in PACo.**

The models tested were as follows: Host; host tracks the symbiont phylogeny, Symbiont; symbiont tracks the host phylogeny, Undetermined; unclear which phylogeny tracks the other, Specialist/generalist symbiont; symbiont tracks the host and the specialist/generalist feature of the symbionts also drive the co-phylogenetic signal. ***; significant pairwise comparisons (Wilcoxon test, *p*-values < 2.22e-16).

**Supplementary Table 1. Summary of haplotypes number and genetic indices per *Harpagifer* host species.**

Number of sequences (*N)*, number of oligotypes (*k)*, number of polymorphic sites (*S*), genetic diversity (*H*), pairwise differences between sequences (Π) and nucleotide diversity (π) are detailed.

**Supplementary Table 2.** **Abundance table of *Harpagifer* haplotypes across sampling localities.**

**Supplementary Table 3. Summary of genetic p-distances (%) within and among the four *Harpagifer* species.**

The genetic p-distances within and among the four species are presented in, and below the diagonal, respectively. The p-values, determined through 10,000 permutations, are shown on the upper diagonal.

**Supplementary Table 4. Pairwise PERMANOVA on gut mucosa microbiome dissimilarities among *Harpagifer* species.**

F-statistics and *p*-values are provided on the lower and upper diagonal, respectively. *P*-values were obtained through 10,000 permutations and were corrected with the Holm method.

**Supplementary Table 5. Mantel test analysis on GMM of *Harpagifer* spp. using weighted UniFrac distances.**

A total of 10,000 permutations were performed. *P*-values in bold are considered as significant (𝜶 = 0.05). For partial mantel, the vertical bar “|” means “controlling for”.
